# Evidence of sound production in wild stingrays

**DOI:** 10.1002/ecy.3812

**Published:** 2022-08-17

**Authors:** Lachlan C. Fetterplace, J. Javier Delgado Esteban, Joni Pini‐Fitzsimmons, John Gaskell, Barbara E. Wueringer

**Affiliations:** ^1^ Department of Aquatic Resources Institute of Coastal Research, Swedish University of Agricultural Sciences Öregrund Sweden; ^2^ Fish Thinkers Research Group Gerroa New South Wales Australia; ^3^ Whale Nation Studio Frigiliana Spain; ^4^ Department of Biological Sciences, Faculty of Science and Engineering Macquarie University Macquarie Park New South Wales Australia; ^5^ Reef Catchments Proserpine Queensland Australia; ^6^ Sharks And Rays Australia Bungalow Queensland Australia

**Keywords:** agonistic display, batoidea, communication, elasmobranch, predator avoidance, sound production

Although almost 990 species of bony fish (Osteichthyes) actively produce sounds, evidence for active sound production by elasmobranchs—sharks, rays, and skates—is scarce (Looby et al., 2022). To date, there have been only 27 examinations of sound production by elasmobranchs (Looby et al., 2022), and of the 13 recorded occurrences, the majority have been passive sounds associated with feeding (e.g., shell crushing; Ajemian et al., 2021). The only confirmed case of active sound production occurred when captive cownose rays *Rhinoptera bonasus* produced short, sharp clicks under duress, that is, forceful prodding (Fish & Mowbray, 1970). Two further examples of “active” sound production have been documented—“crunching” sounds with chewing and “mumbling” after ingestion in a captive common stingray *Dasyatis pastinaca* and “rumbles” when grabbing food by a captive picked dogfish *Squalus acanthias* (Shishkova, 1958)—but these were both associated with feeding and are less convincing.

There have been no confirmed examples of active sound production by elasmobranchs in the wild, despite attempts to record the behavior outside of captive settings. Although there are some anecdotal reports, they remain unproven or are given without sources. Bass and Rice (2010), for example, reported that “stingrays have been anecdotally documented to grind their teeth as an audible defence warning signal,” without providing a reference for this statement. By comparison, the hearing capabilities of elasmobranchs have received much more attention (Mickle et al., 2020; Myrberg, 2001). Elasmobranchs are most sensitive to low‐frequency sounds between 40 and 1500 Hz, with peak sensitivities between 200 and 400 Hz, but audiograms have only been produced for 10 species (Chapuis & Collin, 2022). There is more evidence relating to behavioral responses to sounds. Many sharks are attracted to certain sounds, like those of struggling prey, and can change their behaviors in response to such sounds (Gardiner et al., 2012). Other sounds, such as the vocalizations of killer whales, *Orcinus orca*, reportedly repulse and cause a fleeing response in epipelagic sharks, which could fall prey to these odontocetes (Chapuis et al., 2019; Myrberg, 2001). Similarly, in some shark species an unexpected sound or the sudden increased intensity of a sound can result in rapid withdrawal from the sound source (Klimley & Myrberg, 1979; Myrberg, 2001; Myrberg et al., 1978). Sound may also elicit less obvious responses, for example, the southern stingray *Hypanus americanus* has been shown to alter its swimming behavior (i.e., resting less, increasing swimming activity, and breaching the surface more often) in response to certain sounds (Mickle et al., 2020).

Though it is clear that elasmobranchs can hear and many can also respond to sound in various ways, hearing capacity is not necessarily linked to the ability to produce acoustic sound (Mélotte et al., 2018), and until now there has been limited evidence to suggest that any elasmobranchs have the ability to actively produce sound themselves. Here we present the first records of voluntary active sound production in the wild by three individuals of two species of stingray: the mangrove whipray *Urogymnus granulatus* (Figure 1b) and the cowtail stingray *Pastinachus ater* (Figure 1c). The sounds recorded from all three individuals were characterized by a series of very short, broadband clicks (Figure 1d, Appendix S1: Table S1) and were associated with movement of the spiracles and cranial area. In all recorded observations, the ray commenced producing sounds in response to an observer approaching closely and ceased sound production when the distance between the ray and observer increased. We suggest hypotheses for the potential purposes and mechanisms of the sound production and highlight that further research into this ability is needed.

**FIGURE 1:**
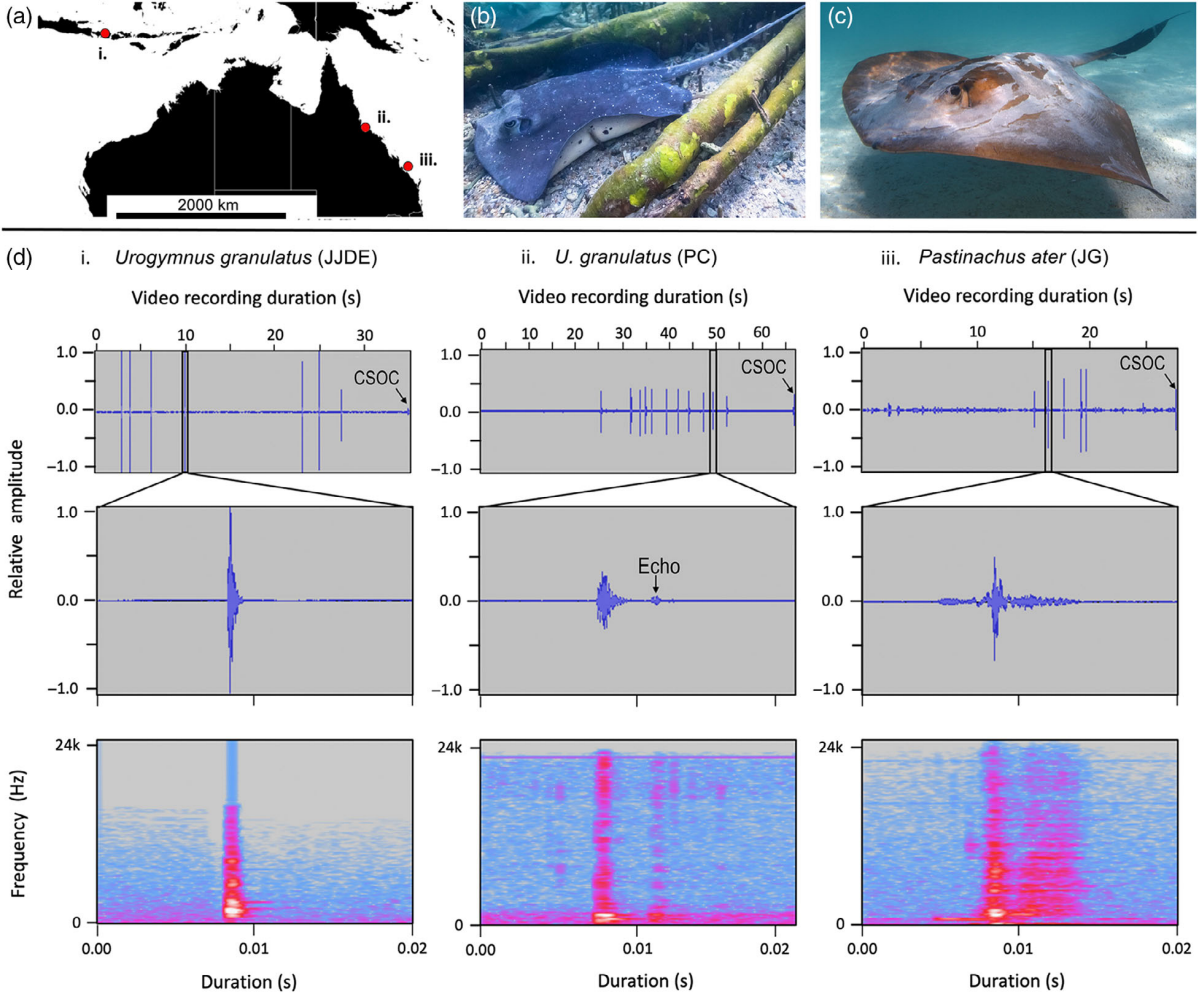
Incidentally recorded observations of sound production by stingrays. (a) Observation locations in waters near (i) Gilli Trawangan, Indonesia (adult *Urogymnus granulatus*, Philip Christoff), (ii) Magnetic Island, Australia (juvenile *U. granulatus*, J. Javier Delgado Esteban), and (iii) Heron Island, Australia (adult *Pastinachus ater*, John Gaskell). (b) A juvenile mangrove whipray *U. granulatus* resting in shallow water, Magnetic Island, Queensland, Australia. Image by J. Javier Delgado Esteban. (c) A cowtail stingray *P. ater* in waters off Heron Island, Queensland, Australia. Image by John Gaskell. (d) Sound profiles of stingray clicks from each observation (i–iii). Top to bottom: Waveforms of each video recording (Video S1) showing all recorded clicks followed by waveforms and spectrograms of a representative click from each video recording (extracted using Audacity version 2.4.2). CSOC, camera shut off click.

The three recorded observations occurred as follows. On 22 December 2018, Philip Christoff (PC) was undertaking a recreational closed‐circuit rebreather dive at the Deep Turbo dive site northeast of Gili Trawangan, Gili Islands, Indonesia (Figure 1a, approximately −8.339491°, 116.048697°). At around 9:30 A.M., PC sighted an adult female mangrove whipray *U. granulatus* (disk width around 1 m) resting under the sand. Following a slow approach by PC, the ray appeared disturbed and slowly swam away parallel to the diver. It began making clicking sounds when PC came within ~2 m. Each click coincided with movement of the spiracle and partial retraction of the eye (Video S1). Eleven broadband clicks were recorded, ranging from 0.017 to 0.025 s in duration (mean ±SE = 0.021 ± 0.001) (Figure 1d‐ii, Appendix S1: Table S1). Clicks 1–10 had a peak frequency of 1500 Hz, and the 11th click had a peak frequency of 1031 Hz (Appendix S1: Table S1). Mean bandwidth (±SE) across all clicks was 22.731 kHz ± 33.883 Hz (Appendix S1: Table S1). Secondary pulses were also noted in the waveforms of each click (Figure 1d‐ii); however, based on their similarity to the primary pulses and lower relative amplitude, these were considered echoes within the camera housing (Nauticam housing on Sony RX100M5 digital camera).

In February 2018, J. Javier Delgado Esteban (JJDE) observed sound production by a juvenile mangrove whipray *U. granulatus* (disk width around 40 cm) while snorkeling in the shallow, inshore waters of Geoffrey Bay, Magnetic Island, Great Barrier Reef, Queensland, Australia (Figure 1a, −19.153243°, 146.867342°). The juvenile was part of a larger group, but it had been separated from the group at the time it was recorded. Seven distinct broadband clicks were observed, ranging from 0.01 to 0.017 s in duration (mean ±SE = 0.013 ± 0.001) (Figure 1d‐i, Appendix S1: Table S1). The first six clicks had a peak frequency of 1687 Hz, but the seventh click had a peak frequency of 1875 Hz (Appendix S1: Table S1). Mean bandwidth (±SE) across all clicks was 22.314 kHz ± 902.754 Hz (Appendix S1: Table S1). The clicks were described as originating from the ventral area of the animal, with each visibly coinciding with contractions of the spiracles (Video S1). Immediately after the sounds were emitted, the rest of the group of stingrays approached both the individual ray and the stationary snorkeler. JJDE observed numerous instances of sound production in this group of juvenile mangrove whiprays over several days, but these were not captured on film.

The third observation was recorded in October 2017 by John Gaskell (JG) when snorkeling with a group of cowtail stingrays *P. ater*, which are known to aggregate in shallow waters off the southern beach of Heron Island (Figure 1a; −23.443510°, 151.913074°), Great Barrier Reef, Queensland, Australia. While filming in water approximately 70 cm deep, JG pursued one animal that was slowly swimming away from him. When JG came within a distance of less than a disk width of the ray, the animal started to produce loud clicking sounds that coincided with contractions of the cranial and spiracle area of the animal (Video S1). Five distinct broadband clicks were recorded, ranging from 0.021 to 0.091 s in duration (mean ±SE = 0.065 ± 0.012) (Figure 1d‐iii, Appendix S1: Table S1). The first click had a peak frequency of 1406 Hz, while clicks 2–5 peaked at 1500 Hz (Appendix S1: Table S1). Mean bandwidth (±SE) across all clicks was 23.904 kHz ± 17.776 Hz (Appendix S1: Table S1). JG observed similar sound production two more times in the same species over 6 days, but these events were not captured on film.

In addition to the foregoing observations recorded on film, in the early 2010s commercial divers from Far North Queensland were hand‐collecting sea cucumbers in the inshore waters of the Great Barrier Reef and Coral Sea up to 20 m in depth and reported that on multiple occasions cowtail stingrays *P. ater*, when approached in murky waters, produced loud clicking sounds while fleeing from divers (B. E. Wueringer, unpublished).

The observed sound production in both species of rays appeared to serve the purpose of agonistic displays. In sharks, agonistic displays are relatively common and mainly comprise visual components, such as lowering of the pectoral fins (silent) or tail slapping or popping, which does produce sounds (Martin, 2007), although these sounds are based on direct observations, and recording and analysis are still needed. In rays, agonistic displays observed to date generally have involved physical intra‐ and interspecific interactions, such as biting, chasing, and shoving (Newsome et al., 2004; Pini‐Fitzsimmons et al., 2021). In contrast, the sudden loud sounds reported here appear to be more likely to represent a warning or serve to startle predators, such as sharks, which have been shown to rapidly flee from sudden unexpected sounds (Klimley & Myrberg, 1979; Myrberg, 2001). Further, since the rays are able to produce these sounds while fleeing from a fight‐or‐flight situation, they do not have to sacrifice their swimming efficiency in order to produce a warning signal (Martin, 2007).

Both juvenile mangrove whiprays *U. granulatus* and cowtail stingrays *P. ater* appear social and are often observed feeding and resting in groups, likely as a predator‐avoidance strategy (Kanno et al., 2019; Martins et al., 2020a, 2020b). In the case of JJDE's observation, other juvenile mangrove whiprays were observed gathering around the individual filmed producing the clicks and appeared to be doing so in response to the produced sounds. Sound production may therefore alert conspecifics to the need to aggregate in response to danger, which also implies a role in intraspecific communication.

The bandwidth of the clicks produced by *U. granulatus* and *P. ater* examined here spanned the expected hearing range of elasmobranchs (40–1500 Hz; Chapuis & Collin, 2022), providing some evidence that their predators (*Carcharhinus melanopterus* and *Negaprion acutidens*; Kanno et al., 2019; Martins et al., 2020a, 2020b) and conspecifics can hear these sounds, although peak frequencies of the clicks occurred at the top or above this hearing range (1031–1875 Hz). However, audiograms have only been produced for a few elasmobranch species, and none has been produced for *U. granulatus*, *P. ater*, or their known predators (Chapuis & Collin, 2022). Further assessment of the hearing abilities of these species is therefore necessary to clarify the role of the produced sounds in agonistic displays or predator avoidance.

The exact mechanism of sound production remains unclear but appears to be similar in both species. In all video recordings, contractions of the spiracles and associated gill openings are visible simultaneously with the clicking sounds (Video S1), indicating that sounds may be produced through fast contractions of the cranial and gill area. Because both species lack myliobatiform grinding plates, which would be positioned on the palate, but instead possess teeth limited to their jaws, the anecdotally proposed mechanism of sound production using grinding plates (Bass & Rice, 2010) is likely incorrect in this instance. Whether the sound production is achieved through fast expulsion of water or another internal mechanism is plausible but awaits verification, and further research on the internal morphology of these rays is required.

The observations presented here highlight that further research on sound production in elasmobranchs is warranted, especially considering the limited number of examinations in this group to date (Looby et al., 2022). Our observations are of species that are encountered relatively often by snorkelers and yet were not previously known to produce sounds. Other similar species may also produce sounds, but anecdotal records may have not yet come to light; thus, our paper may serve to bring to light further examples from the public and researchers. All of the examples presented here were captured opportunistically with handheld digital cameras, and future targeted research should endeavor to use standardized hydrophones (Lindseth & Lobel, 2018; Rountree et al., 2006), where possible, to allow for better control of sound distortion and echoes.

Our observations and Fish and Mowbray's (1970) observations in captivity mean that three ray species (of approximately 245 Myliobatiformes [Stein et al., 2018]) have now been convincingly shown to actively produce sounds and to do so in the wild, voluntarily, and without artificial stimuli. Although elasmobranchs are generally not considered to be sound producing (Looby et al., 2022), our study illustrates that this is a misconception and more research into their ability to produce and hear such sounds is required.

## AUTHOR CONTRIBUTIONS

Video contributions were made by J. Javier Delgado Esteban and John Gaskell. The original draft was written by Barbara E. Wueringer, Lachlan C. Fetterplace, and Joni Pini‐Fitzsimmons, with further review and editing by Barbara E. Wueringer, Lachlan C. Fetterplace, Joni Pini‐Fitzsimmons, and J. Javier Delgado Esteban. Analysis of footage was completed by Joni Pini‐Fitzsimmons. Video S1 was created by John Gaskell.

## CONFLICT OF INTEREST

Authors declare that they have no competing interests.

## Supporting information


Appendix S1

Video S1

Video S1 Legend

## Data Availability

Videographic data (Fetterplace et al., 2022) is provided in Figshare at https://doi.org/10.6084/m9.figshare.16929838.v1.
