# Supplementary material for: Evidence of sound production in wild stingrays
Source: Ecology. 2022 Aug 17;103(11):e3812. doi: 10.1002/ecy.3812 (PMC9786621; doi:10.1002/ecy.3812)
Supplement: Supplementary file 1 — Appendix S1 [file ECY-103-e3812-s002.pdf]

Supporting Information: Fetterplace, L., Esteban, J. J. D., Pini-Fitzsimmons, J., Gaskell, J., Wueringer, B. E. 2022. Evidence of sound production in wild stingrays. Ecology

Appendix S1: Table S1

Table S1. Attributes of sounds produced by adult mangrove whipray *Urogymnus granulatus* (PC), juvenile mangrove whipray *U. granulatus* (JJDE) and adult cowtail stingray *Pastinachus ater* (JG), extracted using Audacity® v2.4.2. See also Video S1.

Observation & click number	Start time (s)	Duration (s)	Peak freq. (Hz)	Min freq. (Hz)	Max freq. (Hz)	Bandwidth (kHz)
Adult *Urogymnus granulatus* (PC)						
1	25.562	0.021	1500	140	22920	22.78
2	32.101	0.017	1500	140	22720	22.58
3	34.059	0.018	1500	140	22850	22.71
4	35.276	0.019	1500	140	22970	22.83
5	36.561	0.019	1500	140	22970	22.83
6	39.736	0.023	1500	140	22920	22.78
7	42.152	0.025	1500	140	22900	22.76
8	44.545	0.022	1500	140	22820	22.68
9	47.699	0.022	1500	140	22920	22.78
10	49.629	0.022	1500	140	22620	22.48
11	52.695	0.021	1031	140	22970	22.83
Juvenile *U. granulatus* (JJDE)						
1	2.861	0.012	1687	800	24000	23.2
2	3.865	0.011	1687	800	24000	23.2
3	6.267	0.011	1687	700	24000	23.3
4	9.971	0.01	1687	800	24000	23.2
5	23.147	0.017	1687	900	24000	23.1
6	25.113	0.017	1687	700	24000	23.3
7	27.551	0.01	1875	900	17800	16.9
Adult *Pastinachus ater* (JG)						
1	15.206	0.021	1406	0	23930	23.93
2	16.399	0.091	1500	0	23930	23.93
3	17.802	0.072	1500	0	23890	23.89
4	19.357	0.071	1500	0	23930	23.93
5	19.794	0.072	1500	0	23840	23.84
