# Supplementary material for: Evidence of sound production in wild stingrays
Source: Ecology. 2022 Aug 17;103(11):e3812. doi: 10.1002/ecy.3812 (PMC9786621; doi:10.1002/ecy.3812)
Supplement: Supplementary file 3 — Video S1 Legend [file ECY-103-e3812-s003.pdf]

Supporting Information: Fetterplace, L., Esteban, J. J. D., Pini-Fitzsimmons, J., Gaskell, J., Wueringer, B. E. 2022. Evidence of sound production in wild stingrays. Ecology

Caption for Video S1

Observations of sound production by stingrays incidentally recorded: adult *Urogymnus granulatus* in Gilli Trawangan, Indonesia (videographer: Philip Christoff), juvenile *U. granulatus* near Magnetic Island, Australia (videographer: J. Javier Delgado Esteban), and adult *Pastinachus ater* near Heron Island, Australia (videographer: John Gaskell). Zoomed in, slow-motion clips of representative clips from each observation are included, showing cranium and spiracle movements associated with sounds produced.
